# The next click in antibody drug conjugate development

**DOI:** 10.1016/j.xcrm.2026.103099

**Published:** 2026-10-20

**Authors:** Avantika Gupta, Sarat Chandarlapaty

**Affiliations:** 1 Human Oncology and Pathogenesis Program, Memorial Sloan Kettering Cancer Center, New York, NY, USA; 2 Department of Medicine, Memorial Sloan Kettering Cancer Center, New York, NY, USA; 3 Weill Cornell Medical College, New York, NY, USA

## Abstract

The clinical success of antibody drug conjugates has been shaped by the promise of delivering cytotoxic payloads precisely to tumors while minimizing systemic exposure. Simó and colleagues aim to improve on conventional ADC delivery by decoupling tumor localization from payload delivery to enhance pharmacokinetics and addressing antigen heterogeneity by enabling dual targeting.

## Main text

Antibody drug conjugates (ADCs) have emerged as one of the most powerful therapeutic platforms in oncology, combining the tumor-targeting precision of monoclonal antibodies with the cytotoxic potential of small molecule payloads. Early ADCs were limited by heterogeneous conjugation chemistry, unstable linkers, and payloads with narrow therapeutic windows, but advances across all three components have resulted in a new generation of highly active agents. Development of optimized linker-payload systems and membrane-permeable payloads capable of killing neighboring antigen-negative cells through bystander activity have expanded the therapeutic reach of ADCs beyond the antigen-high populations originally targeted. The clinical validation of this approach spans multiple tumor types and target antigens, with approved ADCs now directed against HER2, TROP2, FRα, Nectin-4, and others, demonstrating that the platform is broadly applicable rather than antigen- or histology-specific. This success has spawned a dramatic shift in drug development with much greater appreciation for the role of the delivery vehicle while simultaneously inspiring efforts to build upon this foundation and address the limitations that remain.

A key limitation of currently utilized antibody formats is that less than 1% of the injected dose reaches the tumor, with the remainder distributed across normal tissues and eliminated through metabolic pathways that generate systemic free payload exposure.^1^ This explains why ADC toxicity profiles can resemble those of conventional chemotherapies. Linker instability in circulation compounds this further, releasing payload prematurely and reducing the amount of intact ADC available for tumor-directed delivery. In addition to toxicity concerns, reliance on a single antibody target provides the tumor an escape mechanism in which acquired resistance can arise through binding site mutations that reduce antibody affinity, transcriptional or epigenetic silencing of target expression, and spatial heterogeneity in antigen expression across tumor subclones.^2^^,^^3^ Beyond antigen loss, the trafficking of the antibody-antigen complex after internalization is a critical and underappreciated determinant of efficacy, governed by antigen density, membrane compartmentalization, and the presence of dimerization partners that can divert the receptor-ADC complex away from productive lysosomal delivery.^4^^,^^5^

Several strategies have been explored to address the limits of the initial single antigen targeting ADCs. Combinations of unconjugated antibodies with ADCs have leveraged existing clinical agents to restore internalization or block compensatory signaling pathways that divert receptor-ADC complexes away from lysosomal trafficking.^6^ Antigen loss and expression heterogeneity have been approached through biparatopic and bispecific ADCs, extending dual engagement from a single antigen to distinct antigens to enforce internalization and provide simultaneous coverage of antigenically heterogeneous tumors in a single molecule.^7^^,^^8^ However, engineering bispecific activity into a single molecule introduces trade-offs where the large molecular size can restrict tumor penetration in dense solid tumors and lead to unpredictable clearance reducing the fraction reaching the disease site. Moreover, these agents risk off-target toxicity in healthy tissues that express even low levels of either target antigen.^9^

To address these challenges, Simó and colleagues introduce a modular *in vivo* click chemistry platform that enables *in vivo* assembly of dual-targeting antibody-ADC constructs.^10^ The approach exploits inverse electron-demand Diels-Alder cycloaddition between *trans*-cyclooctene and tetrazine handles conjugated to separate antibodies, administered sequentially with a 24-h interval between doses. The first antibody is distributed with normal IgG pharmacokinetics, and then the second antibody, which carries the cytotoxic payload, clicks covalently onto the first upon administration. The authors postulate that the majority of the ligation happens at the tumor site, where the tumor receptor biology can also aid with crosslinking and internalization, but a fraction of ligation between the antibody and ADC can also occur during circulation. The central advance is that sequential *in vivo* assembly preserves the favorable biodistribution of individual IgG molecules, generating functional dual-targeting activity at the tumor site while reducing, though not eliminating, the fraction of active conjugate circulating in normal tissues. In theory, this increases the proportion of payload-tumor interaction relative to pre-formed bispecific constructs, which are subject to higher systemic clearance before reaching the disease site. The platform could simultaneously address multiple resistance mechanisms, such as antigen heterogeneity through dual targeting, internalization defects through receptor crosslinking, and pharmacokinetic limitations through staged biodistribution. The authors demonstrate these proposed advantages elegantly in an admixed breast tumor model containing both HER2-expressing and HER2-negative cells where the *in vivo* click combination produced significant tumor growth inhibition (96% click vs. 85.5% no-click tumor growth inhibition on day 13, T-DXd no click x click, *p* = 0.0018) and improved survival compared to the same antibody or ADC administered without click-mediated ligation.

Several questions emerge for this concept to be realized as a clinical advance, including elucidating the specific mechanism(s) of enhanced internalization, the generalizability of the efficacy benefit across distinct antigen pairings, and the regulatory implications of each conjugated antibody constituting a new molecular entity. Future work will need to address these gaps in addition to multi-dose efficacy and toxicity studies in humanized models. Nevertheless, the conceptual framework introduced here of assembling therapeutic specificity *in vivo* rather than engineering it into a fixed molecular structure represents a distinct addition to the growing arsenal of strategies directed at advancing the ADC platform. As the field continues to tackle the complexity of tumor heterogeneity, imperfect payload delivery, and the limits of single-agent targeting, molecules that offer modularity and adaptability without sacrificing pharmacokinetic integrity may prove to be an important iteration on chemical advances in drug delivery driving improvements in therapeutic outcomes.

## Declaration of interests

S.C. has received institutional grant funding from AstraZeneca and Pathos.ai; and received honoraria as a consultant to Daiichi-Sankyo, AstraZeneca, Novartis, Astellas, Bayer, Casdin Capital, Merck, Nuvalent, and Pathos.ai.
